# Cardiopulmonary Resuscitation: Let’s Together Step into a New Era!

**DOI:** 10.3390/jpm12111825

**Published:** 2022-11-02

**Authors:** Wataru Takayama, Yasuhiro Otomo

**Affiliations:** 1 Department of Acute Critical Care and Disaster Medicine, Graduate School of Tokyo Medical and Dental University, 1-5-45, Yushima, Bunkyo-ku, Tokyo 113-0034, Japan; 2 Trauma and Acute Critical Care Center, Tokyo Medical and Dental University Hospital of Medicine, 1-5-45, Yushima, Bunkyo-ku, Tokyo 113-0034, Japan

## 1. Introduction

Although the out-of-hospital cardiac arrest (OHCA) survival rate has improved due to the spread of cardiopulmonary resuscitation (CPR) techniques and insights, OHCA remains a major public health issue worldwide [1,2,3].

In September 2022, our Special Issue titled “Cardiopulmonary Resuscitation: Let’s Together Step into a New Era!” was launched in the Journal of Personalized Medicine. Several articles on various topics in CPR will be published, including new studies on treatment. We introduce these papers below and encourage frontier papers on novel findings for the subsequent series. Our journal could provide useful information for improving clinical outcomes after adult OHCA.

## 2. Influence of Coronavirus Disease 2019 

The unprecedented coronavirus disease 2019 (COVID-19) pandemic continues, and the outcomes and clinical characteristics of OHCA due to direct or indirect pandemic effects have received attention. Some meta-analyses reported implications for OHCA epidemiological features and mortality, which differed significantly from those before the pandemic [4,5]. Several studies found that the composition of patients with OHCA changed, with the number of cardiac arrests increasing in COVID-19 patients with respiratory failure. Furthermore, an increase in arrests in private spaces was found to be an indirect effect of social distancing, self-isolation, and the weakening of the survival chain owing to the deviation of medical resources towards COVID-19. However, many factors have changed, including the systematic medical environment, the ability of patients to respond to confirmed or suspected COVID-19, the vaccination rate for COVID-19 [6], the primary COVID-19 subtype [7], and the recent environmental region of COVID-19 spread. Therefore, because these factors were found to have a direct or indirect effect on OHCA in previous studies, a more stratified analysis is required in the constantly changing pandemic environment.

## 3. Extracorporeal CPR

Several studies [8,9], including small, randomized controlled trials [10], have demonstrated that patients with OHCA who receive extracorporeal CPR (ECPR) have better outcomes than those who receive traditional resuscitation, including increased survival following hospital discharge and improved neurological outcomes.

However, ECPR requires several human and healthcare resources; these are costly and technically challenging, making it difficult to establish programs. 

To establish an ECPR program, the following three broad categories should be considered: (1) eligible patient selection, including patient- and cardiac arrest-specific factors; (2) the duration of the cardiac arrest, including transport time; and (3) location, including whether the receiving hospital could expeditiously provide ECPR. 

Previous protocols and guidelines strived to identify patients most likely to survive with favorable neurological outcomes; these included an age of <70 years, witnessed arrest, an arrest to first CPR (no-flow interval) of <5 min, an initial cardiac rhythm of ventricular tachycardia and fibrillation, and cardiogenic arrest [11,12]. However, no robust data are available to identify those who may benefit from ECPR. Although it has been reported that most survivors underwent extracorporeal membrane oxygenation support within <60 min of the onset of cardiac arrest, among non-hypothermic arrests, the optimal duration before the initiation of ECPR in cases of refractory cardiac arrest is yet to be determined [13]. Many hospitals have specific time-based criteria, including the no-flow time (the time from the onset of cardiac arrest to starting CPR) and low-flow time (the time from CPR to ECPR cannulation), highlighting the need to transport ECPR candidates to appropriate centers [14,15].

Therefore, understanding the current and future state of ECPR is a critical step toward effective worldwide implementation. Additionally, further research into economic analyses, including cost-effectiveness and cost-utility, is required before the global adoption of ECPR programs.

In this upcoming Special Issue of the Journal of Personalized Medicine, we aim to discuss the etiology, pathophysiology, clinical manifestations, diagnostics, and optimal management involved in treating patients with OHCA. Since we aim to attract a global audience, we welcome contributions on the subject from anywhere in the world.

## References

[1] Donoghue A., Hsieh T.C., Myers S., Mak A., Sutton R., Nadkarni V. (2015). Videographic assessment of cardiopulmonary resuscitation quality in the pediatric emergency department. Resuscitation.

[2] Kitamura T., Iwami T., Kawamura T., Nagao K., Tanaka H., Hiraide A., Implementation Working Group for the All-Japan Utstein Registry of the Fire and Disaster Management Agency (2010). Nationwide public-access defibrillation in Japan. N. Engl. J. Med..

[3] Ambulance Service Planning Office of Fire and Disaster Management Agency of Japan Effect of First Aid for Cardiopulmonary Arrest. https://www.fdma.go.jp/neuter/topics/kyukyukyujo_genkyo/h28/01_kyukyu.pdf.

[4] Teoh S.E., Masuda Y., Tan D.J.H., Liu N., Morrison L.J., Ong M.E.H., Blewer A.L., Ho A.F.W. (2021). Impact of the COVID-19 pandemic on the epidemiology of out-of-hospital cardiac arrest: A systematic review and meta-analysis. Ann. Intensive Care.

[5] Bielski K., Szarpak A., Jaguszewski M.J., Kopiec T., Smereka J., Gasecka A., Wolak P., Nowak-Starz G., Chmielewski J., Rafique Z. (2021). The influence of COVID-19 on out-hospital cardiac arrest survival outcomes: An updated systematic review and meta-analysis. J. Clin. Med..

[6] Jabłońska K., Aballéa S., Toumi M. (2021). The real-life impact of vaccination on COVID-19 mortality in Europe and Israel. Public Health.

[7] Maslo C., Friedland R., Toubkin M., Laubscher A., Akaloo T., Kama B. (2022). Characteristics and outcomes of hospitalized patients in South Africa during the COVID-19 Omicron wave compared with previous waves. J. Am. Med. Assoc..

[8] Sakamoto T., Morimura N., Nagao K., Asai Y., Yokota H., Nara S., Hase M., Tahara Y., Atsumi T., SAVE-J Study Group (2014). Extracorporeal cardiopulmonary resuscitation versus conventional cardiopulmonary resuscitation in adults with out-of-hospital cardiac arrest: A prospective observational study. Resuscitation.

[9] Inoue A., Hifumi T., Sakamoto T., Kuroda Y. (2020). Extracorporeal cardiopulmonary resuscitation for out-of-hospital cardiac arrest in adult patients. J. Am. Heart Assoc..

[10] Yannopoulos D., Bartos J., Raveendran G., Walser E., Connett J., Murray T.A., Collins G., Zhang L., Kalra R., Kosmopoulos M. (2020). Advanced reperfusion strategies for patients with out-of-hospital cardiac arrest and refractory ventricular fibrillation (ARREST): A phase 2, single centre, open-label, randomised controlled trial. Lancet.

[11] Donnino M.W., Andersen L.W., Deakin C.D., Berg K.M., Böttiger B.W., Callaway C.W., Drennan I., Neumar R.W., Nicholson T.C., O’Neil B.J. (2018). Extracorporeal Cardiopulmonary Resuscitation (ECPR) for Cardiac Arrest—Adults Consensus on Science with Treatment Recommendations [Draft].

[12] Yannopoulos D., Bartos J.A., Aufderheide T.P., Callaway C.W., Deo R., Garcia S., Halperin H.R., Kern K.B., Kudenchuk P.J., Neumar R.W. (2019). The evolving role of the Cardiac Catheterization Laboratory in the management of patients with out-of-hospital cardiac arrest: A scientific statement from the American Heart Association. Circulation.

[13] Bartos J.A., Grunau B., Carlson C. (2019). Improved survival with extracorporeal life support despite progressive metabolic derangement associated with prolonged resuscitation. J. Am. Coll Cardiol..

[14] Otani T., Sawano H., Natsukawa T., Nakashima T., Oku H., Gon C., Takahagi M., Hayashi Y. (2018). Low-flow time is associated with a favorable neurological outcome in out-of-hospital cardiac arrest patients resuscitated with extracorporeal cardiopulmonary resuscitation. J. Crit. Care.

[15] Matsuyama T., Irisawa T., Yamada T., Hayakawa K., Yoshiya K., Noguchi K., Nishimura T., Ishibe T., Yagi Y., Kiguchi T. (2020). Impact of low-flow duration on favorable neurological outcomes of extracorporeal cardiopulmonary resuscitation after out-of-hospital cardiac arrest: A multicenter prospective study. Circulation.

